# Web-based interventions for traumatized people in mainland China

**DOI:** 10.3402/ejpt.v5.26519

**Published:** 2014-12-09

**Authors:** Jian-Ping Wang, Andreas Maercker

**Affiliations:** 1 School of Psychology, Beijing Normal University, Beijing, People's Republic of China; 2 Department of Psychiatry and Psychotherapy, University of Zurich, Zürich, Switzerland

**Keywords:** Internet interventions, online self-help, e-health, CBT, RCT, efficacy

## Abstract

**Background:**

The Internet is now becoming a new channel for delivering psychological interventions.

**Method:**

This paper reported a first application of web-based intervention in mainland China. It first summarized primary barriers to mental health help-seeking behavior in Chinese society. Then, it introduced the current utilization of the Internet within mental health services in mainland China and discussed how the Internet would help to improve people's help-seeking behaviors. More importantly, it presented main empirical findings from a randomized controlled trial (RCT) which investigated the efficacy of a web-based self-help intervention program (Chinese My Trauma Recovery website, CMTR) for 103 urban and 93 rural traumatized Chinese persons.

**Results:**

The data revealed that 59% urban and 97% rural participants completed the posttest. In the urban sample, data showed a significant group×time interaction in Posttraumatic Diagnostic Scale (PDS) scores (F1,88=7.65, *p*=0.007). CMTR reduced posttraumatic symptoms significantly with high effect size after intervention (F1,45=15.13, Cohen's *d*=0.81, *p*<0.001) and the reduction was sustained over a 3-month follow-up (F1,45=17.29, Cohen's *d*=0.87, *p*<0.001). In the rural sample, the group×time interaction was also significant in PDS scores (F1,91=5.35, *p*=0.02). Posttraumatic symptoms decreased significantly after intervention (F1,48=43.97, Cohen's *d*=1.34, *p*<0.001) and during the follow-up period (F1,48=24.22, Cohen's *d*=0.99, *p*<0.001).

**Conclusions:**

These findings give preliminary support for the short-term efficacy of CMTR in the two Chinese populations. Finally, some implications are given for the future application of web-based interventions for PTSD in mainland China.

